# The impact of a produce prescription programme on healthy food purchasing and diabetes-related health outcomes

**DOI:** 10.1017/S1368980021001828

**Published:** 2021-08

**Authors:** Julian Xie, Ashley Price, Neal Curran, Truls Østbye

**Affiliations:** 1 Duke University, School of Medicine, Durham, NC, USA; 2 Duke University, Department of Family Medicine and Community Health, Suite 400, 2200 W. Main Street, Durham, NC27705, USA; 3 Reinvestment Partners, Durham, NC, USA

**Keywords:** Produce Prescription Programme, Nutrition incentive, Clinic community partnership, Food insecurity, Grocery store transaction data, Electronic health records

## Abstract

**Objective:**

To evaluate a Produce Prescription Programme’s utilisation and its effects on healthy food purchasing and diabetes control among participants with type 2 diabetes.

**Design:**

Prospective cohort study using participants’ electronic health records and food transaction data. Participants were categorised as ‘Frequent Spenders’ and ‘Sometimes Spenders’ based on utilisation frequency. Multivariate regressions assessed utilisation predictors and programme effects on fruit/vegetable purchasing (spending, expenditure share and variety) and on diabetes-related outcomes (HbA1c, BMI and blood pressure).

**Setting:**

Patients enrolled by clinics in Durham, North Carolina, USA. Participants received $40 monthly for fruits and vegetables at a grocery store chain.

**Participants:**

A total of 699 food-insecure participants (353 with diabetes).

**Results:**

Being female and older was associated with higher programme utilisation; hospitalisations were negatively associated with programme utilisation. Frequent Spender status was associated with $8·77 more in fruit/vegetable spending (*P* < 0·001), 3·3 % increase in expenditure share (*P* = 0·007) and variety increase of 2·52 fruits and vegetables (*P* < 0·001). For $10 of Produce Prescription Dollars spent, there was an $8·00 increase in fruit/vegetable spending (*P* < 0·001), 4·1 % increase in expenditure share and variety increase of 2·3 fruits/vegetables (*P* < 0·001). For the 353 participants with diabetes, there were no statistically significant relationships between programme utilisation and diabetes control.

**Conclusions:**

Programme utilisation was associated with healthier food purchasing, but the relatively short study period and modest intervention prevent making conclusions about health outcomes. Produce Prescription Programmes can increase healthy food purchasing among food-insecure people, which may improve chronic disease care.

## Graphical abstract

**Figure:**
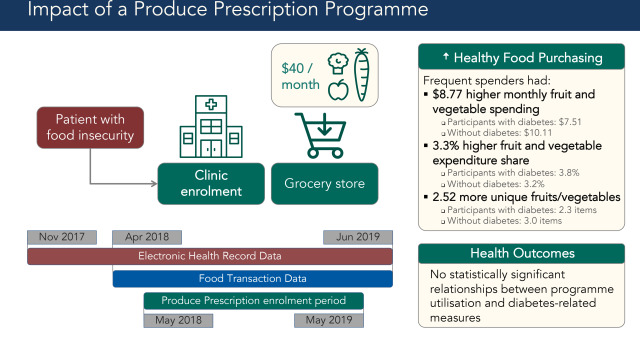

Equitable access to healthy food remains an unmet need in North Carolina, as in much of the United States. Structural inequities create food environments with few healthy options and abundant processed food and fast food^(1)^. North Carolina is consistently one of the most food-insecure states in the country, ranking tenth highest in 2018^(2,3)^. In Durham, 13·5 % of Durham residents were food insecure and one-third of adults had obesity in 2018, and ‘Obesity, Diabetes, and Food Access’ is among residents’ top health priorities^(4)^.

The Supplemental Nutrition Assistance Program (SNAP) helps low-income households meet basic food needs. However, SNAP beneficiaries often have lower diet quality because of economic pressure to stretch benefits by purchasing energy-dense but nutrient-poor foods^(5)^. Thus, food insecurity can cause and exacerbate chronic disease despite existing food assistance resources^(6)^. Nutrition incentive programmes attempt to address this problem by subsidising healthy food purchases. Thus, nutrition incentive programmes increase purchasing power for food and promote healthy food intake among low-socio-economic status individuals^(7)^.

To further build upon nutrition incentive programmes, community-based organisations and health centres have invested in Produce Prescription Programmes. In these programmes, healthcare workers identify food-insecure patients and ‘prescribe’ monetary incentives to purchase fruits and vegetables, or healthy food provided onsite in health settings^(8)^. These innovative programmes incorporate food access directly into the patient–provider relationship, with the intent of promoting healthy eating by better-enabling patients to follow healthcare providers’ dietary advice. Produce Prescription Programmes are relatively new, reaching prominence in the 2010s through various pilot programmes and new US government funding initiatives under the 2014 and 2018 Farm Bills^(9)^.

To date, Produce Prescription Programme research has often focused on small pilot programmes with limited use of food transaction and health data^(9)^. A scoping review of 186 Food as Medicine interventions found that 72 % of interventions and retail nutrition programmes were effective at achieving one or more of the outcomes reported, with the most effective being multi-component interventions combining price incentives, nutrition incentives and marketing nudges. However, the review points to a notably low number of studies on how Food as Medicine programmes interact with specific disease diagnoses and health outcomes. Only twenty of 186 studies focused on populations with specific diseases; ten of these targeted diabetes^(10)^.

We evaluated a new Durham Produce Prescription Programme facilitated by the non-profit organisation Reinvestment Partners. This programme gave food-insecure patients $40 monthly to spend on fruits and vegetables at a major grocery store chain. Our study is novel in its integration of food transaction data and electronic health records (EHR). The analytic sample included participants whose food transaction data could be matched to their EHR. Our study aimed to (1) assess predictors of programme utilisation; (2) assess the association between programme utilisation and healthy food purchasing and (3) explore the association between programme utilisation and diabetes control among the participant subsample with type 2 diabetes.

## Methods

### The intervention

Healthcare workers identified food insecure patients, using screening tools such as the Hunger Vital Sign or their informal understanding of patients’ socio-economic situations, and enrolled them into the Produce Prescription Programme using an online portal between May 2018 and June 2019^(11)^. Patients were eligible if they received SNAP and were over 18 years old. Enrolment required a partner grocery store chain loyalty card; patients without a loyalty card acquired one as part of enrolment. Enrolment sites included a federally qualified health centre, an academic outpatient clinic serving low-income patients and two organisations that refer seniors to health-related social services. Social workers, case managers, dietitians and medical students enrolled patients during clinic visits, as well as through the clinics’ group diabetes education classes. Participants received an instructional handout after enrolment.

Once enrolled, participants received $40 monthly for up to 1 year to spend at partner grocery store chain locations in Durham. Produce Prescription Dollars could be spent on Women, Infants and Children programme-approved fruits and vegetables. Eligible items included fresh, frozen or canned fruit, vegetables and beans without added salt, sugar or fat. Participants received $40 monthly contingent on purchasing at least one item at a partner grocery store using their SNAP Electronic Benefit Transfer card and store loyalty card. Produce Prescription Dollars were applied automatically as the tender (payment method) of first resort for eligible items.

The Produce Prescription Programme evaluated in the current study was funded by the US Department of Agriculture Food Insecurity Nutrition Incentive programme, and the current study was funded by Blue Cross Blue Shield of North Carolina Foundation.

### Data sources

**Fig. 1:**
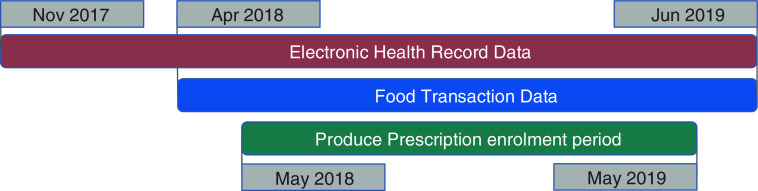
Data timeline for Produce Prescription Programme. Timeline for electronic health records (her), food transaction and Produce Prescription Programme enrolment data. Diagram showing dates of availability for each data source. Red rectangle shows EHR data from November 2017 to June 2019, a blue rectangle shows food transaction data from April 2018 to June 2019 and a green rectangle shows the Produce Prescription enrolment period from May 2018 to May 2019

The study sample included all Produce Prescription participants for whom it was possible to match their programme administration data, grocery store food transaction data and electronic health records (EHR) data. The Duke Health Institutional Review Board approved a waiver of consent for analysis of secondary EHR and programme administration data. We acquired grocery store food transaction data from April 2018 to June 2019, or 1 month prior to the first participant enrolment (Fig. 1). We accessed EHR from November 2017 to June 2019, enabling health data analysis from 6 months before enrolment through the end of programme participation.

From programme administration data, we acquired enrolment date and location and loyalty card number. From EHR, we acquired demographic information, including age, sex and race or ethnicity. We also measured healthcare system utilisation and baseline health status through clinic visits and hospitalisations documented in EHR. From food transaction data, we accessed food item barcodes, descriptions, prices and payment methods. Through payment method, we could determine when participants spent Produce Prescription Dollars. Food transaction data were linked to the participant by loyalty card number.

To merge these data sources, we matched names and phone numbers to medical record numbers, enabling matching of loyalty card numbers to medical record numbers. Using loyalty card numbers and medical record numbers, we merged food transaction data with EHR. We then merged food transaction data with proprietary barcode databases, which enabled food transaction categorisation.

### Measures

To assess predictors of programme utilisation (Aim 1), we measured programme utilisation in two ways. First, we measured it as a binary outcome, where utilisation was defined as having spent Produce Prescription Dollars at least once following enrolment. Second, we calculated a ‘utilisation ratio’ equal to months in which a participant spent Prescription Dollars divided by months with food transaction data available for that participant.

We used the utilisation ratio to categorise those who used the programme at least once into: (1) Frequent Spenders who either spent Produce Prescription Dollars every month or were in the fourth quartile for utilisation ratio and (2) Sometimes Spenders who spent Prescription Dollars at least once up to the fourth quartile cut-off for utilisation ratio. This categorisation was chosen due to programme utilisation’s non-normal distribution and the lack of reliable food transaction data for those who never spent Prescription Dollars after enrolment.

To evaluate effects on healthy food purchasing (Aim 2), we used fruit and vegetable purchasing as the main outcome, and we used three measures similar to those in previous studies^(12)^. First, we used monthly fruit and vegetable spending: dollars spent on fruits or vegetables, including those not eligible for purchase using Produce Prescription Dollars for a more complete approximation of participants’ consumption. Second, we calculated monthly fruit and vegetable expenditure share, or fruit and vegetable spending as a proportion of total food spending. Third, we measured fruit and vegetable diversity by counting the unique fruit and vegetable items purchased in a month.

To assess effects on diabetes-related outcomes (Aim 3), we calculated quarter averages for HbA1c (HbA1c), BMI and systolic blood pressure. A participant’s first quarter average was calculated using data from a time period 3 months before their individual programme enrolment date. We then calculated averages of the health measures for each quarter thereafter, up to four quarters after enrolment. All quarterly averages were calculated at the individual level, recognising participants could enrol on different dates. We set the first post-enrolment quarter 1 month after enrolment to account for potential lag in programme utilisation and delayed effects on health measures (i.e. accounting for the fact that a participant could enrol within one calendar month but not start spending until the following calendar month).

We also analysed EHR data to acquire demographic and health utilisation measures. We assigned primary health insurance by finding the most commonly listed payor among each participant’s healthcare encounters. Health utilisation was measured through outpatient clinic visits, emergency department (ED) visits that did not result in an inpatient admission and hospitalisations in the year following enrolment. If multiple healthcare encounters occurred on the same day, we only counted one of each type at most.

### Statistical analysis

For predictors of programme utilisation (Aim 1), we used *χ* ^2^ tests and ANOVA to assess whether utilisation differed by demographics, health insurance, enrolment site, baseline health status and healthcare utilisation. We used logistic regression with programme utilisation as a discrete outcome. We also conducted logistic regression with Frequent Spender status as a binary outcome relative to Sometimes Spender status.

To assess how programme utilisation affected healthy food purchasing (Aim 2), we performed multivariable random effects panel regression to assess monthly fruit and vegetable purchasing. For each of the three fruit and vegetable purchasing outcome measures, we tested models with programme utilisation measured by Frequent Spender status or monthly Produce Prescription Dollars spent as the key explanatory variable. For Aim 2, we conducted analyses on the full sample with diabetes diagnosis as one of the covariates. We subsequently also conducted analyses on participants with and without diabetes to compare programme effects for these two groups.

To evaluate how programme utilisation affected diabetes-related outcomes (Aim 3), we used multivariable panel regression with 3-month averages of HbA1c, BMI and systolic blood pressure. The key explanatory variables for these models were Frequent Spender status.

For Aims 2 and 3, we included the following potential confounders: demographics (race or ethnicity, sex and age), health insurance, enrolment site, healthcare utilisation (outpatient clinic visits, ED visits and hospitalisations), month-fixed effects and the maximum possible months the participant could have utilised the programme (accounting for potential programme experience differences between early and late participants). For Aim 2, we additionally controlled for non-fruit and vegetable spending as a proxy for household size.

## Results

### Subject characteristics

This Produce Prescription Programme enrolled 807 patients and we matched 699 participants’ food transaction data and EHR. Data were not available on how many patients who were offered programme enrolment. The majority of participants were Black (81 %), elderly (52 % were 60 or older) and female (72 %) (Table 1).

**Table 1:** Subject characteristics and predictors of utilisation for a produce prescription programme in Durham, NC, 2018–2019

Category	Overall		Distribution by spender type‡						Multivariate logistic regression§	
	*n*	%	Never spender		Sometimes spender		Frequent spender		OR	95 % CI
Participants	699		118		339		242			
	Mean	sd	Mean	sd	Mean	sd	Mean	sd		
Average age (years)**	58·5	14·1	53·3	15·3	59·1	13·7	60·1	13·6	1·03	1·01, 1·05†
Sex**										
Female	506	72·4	13·6 %		51 %		35·4 %		Base category	
Male	193	27·6	25·4 %		42 %		32·6 %		0·45	0·28, 0·73††
Race or ethnicity*										
Black	569	81·4	15·1 %		48·7 %		36·2 %		Base category	
White non-Hispanic	81	11·6	22·2 %		43·2 %		34·6 %		0·70	0·35, 1·38
Hispanic (regardless of race)	37	5·3	27 %		62·2 %		10·8 %		0·59	0·22, 1·58
Other	12	1·7	33·3 %		33·3 %		33·3 %		0·49	0·12, 1·98
Enrolment location*										
FQHC	300	42·9	21 %		46·3 %		32·7 %		0·58	0·21, 1·61
Senior assistance organisations	194	27·8	8·3 %		51 %		40·7 %		1·35	0·44, 4·13
Academic primary care clinic	122	17·5	18 %		48·4 %		33·6 %		0·72	0·24, 2·16
Dept of health	39	5·58	25·6 %		46·2 %		28·2 %		0·31	0·090, 1·08
Other	44	6·29	15·9 %		54·6 %		29·6 %		Base category	
Primary insurance*										
Medicare	153	21·9	11·8 %		47·1 %		41·2 %		0·87	0·39, 1·93
Medicare advantage	137	19·6	14·6 %		48·9 %		36·5 %		0·59	0·26, 1·35
Medicaid	169	24·2	19·5 %		44·4 %		36·1 %		1·03	0·53, 2·01
Private	92	13·2	13·0 %		50·0 %		36·1 %		1·05	0·46, 2·41
Other: uninsured, VA, or unlisted	148	21·2	23·7 %		53·4 %		23·0 %		Base category	
Health status and healthcare utilisation										
Diabetes diagnosis	353	50·5	14·2 %		51·0 %		34·8 %		1·42	0·87, 2·33
Average BMI, pre-enrolment 6 months (kg/m^2^)‖	33·8	9·42	32·2	10·0	33·8	9·08	34·5	9·63	1·01	0·99, 1·04
Average systolic blood pressure, pre-enrolment 6 months (mmHg)¶	130·5	16·5	129·9	19·3	131·0	16·0	130·2	15·7	0·99	0·98, 1·01
Average clinic visits, year post-enrolment	14·4	14·2	12·2	13·1	15·1	15·6	14·6	12·5	1·03	1·00, 1·05†
Average emergency department visits, year post-enrolment*	1·35	3·01	1·86	3·83	1·41	3·41	1·03	1·63	0·94	0·88, 1·01
Average hospitalisations, year post-enrolment	0·49	1·18	0·53	1·21	0·54	1·27	0·40	1·04	0·81	0·75, 0·98†

* Categories with statistically significant ANOVA or *χ* ^2^ test (*P* < 0·05), indicating difference between Never-Spenders, Sometimes Spenders and Frequent Spenders.

† Statistically significant coefficient (*P* < 0·05) for multivariate logistic regression with ever-utilising programme as binary outcome.

‡ Continuous variables are presented as Mean (sd), while categorical variables show row percentages (i.e. proportion within each spender type).

§ *n* 607 for multivariate logistic regression.

‖ *n* 609.

¶ *n* 610.

** Categories with *P* < 0·01.

†† Coefficient with *P* < 0·01.

All other *n* 699 unless specified.

The average utilisation ratio among those who spent Produce Prescription Dollars at least once was 73 %. To distinguish Frequent Spenders from Sometimes Spenders, we excluded people who never utilised the programme and then used the fourth quartile cut-off for utilisation ratio, which was 87·5 %. There were 242 Frequent Spenders and 339 Sometimes Spenders. For analyses on healthy food purchasing (Aim 2) and diabetes-related outcomes (Aim 3), we excluded the 119 ‘Never Spenders’ since their spending at our partner grocery stores was so low that their food transaction data were unlikely to represent their consumption accurately.

Further, we conducted a sensitivity analysis including only the 322 participants with food transaction data available before enrolment, meaning they already had a store loyalty card. We expected these individuals’ food transaction data to accurately represent their overall food purchase and consumption. Within this sensitivity analysis, the results were similar, so we retained all 581 participants who ever redeemed Produce Prescription Dollars for the analyses in Aim 2 and Aim 3.

### Aim 1

#### Predictors of programme utilisation

Table 1 shows the association of demographic characteristics and healthcare utilisation patterns with programme utilisation as a binary outcome, as well as individual *χ* ^2^ or ANOVA tests for each characteristic. Higher programme utilisation frequency was associated with older age, female sex, Black race, enrolment through senior assistance organisations and fewer ED visits. Participants with Medicare, Medicare Advantage or private insurance were also comparatively more likely to utilise the programme more frequently.

In the multivariate logistic regression also shown in Table 1, higher programme utilisation likelihood was associated with female sex (male sex OR = 0·45, 95 % CI 0·28, 0·73), older age (OR = 1·03, 95 % CI 1·01, 1·05) and more outpatient clinic visits (OR = 1·03, 95 % CI 1·00, 1·05). A higher number of hospitalisations (OR = 0·81, 95 % CI 0·75, 0·98) were associated with *lower* programme utilisation likelihood.

In an alternative multivariate logistic regression of Frequent Spender *v*. Sometimes Spender status, more frequent utilisation was associated with Medicare (OR = 2·06, 95 % CI 1·06, 4·31), Medicare Advantage (OR = 1·88, 95 % CI 1·00, 3·54) and Medicaid (OR = 1·75, 95 % CI 1·03, 2·98). In both multivariate regressions, programme utilisation was otherwise not significantly associated with other health status and utilisation measures, including diabetes diagnosis, BMI, systolic blood pressure or ED visits.

Table 2 shows a comparison between participants with and without a diabetes diagnosis. Participants with diabetes had a higher average age, which was also evidenced through a higher representation of Medicare enrolees and recruitment from the two senior assistance organisations. Participants with diabetes also had higher baseline HbA1c, BMI and systolic blood pressure and more frequent outpatient clinic visits and hospitalisations.

**Table 2:** Comparison between individuals with diabetes diagnosis (*n* 353) and without diabetes diagnosis (*n* 346) participating in a produce prescription programme, Durham, NC, 2018–2019

Category	Participants without diabetes diagnosis		Participants with diabetes diagnosis	
	Mean	sd	Mean	sd
Average age* (years)	55·6	15·8	61·2	11·6
	*n*	%	*n*	%
Sex				
Female	252	72·8	254	72·0
Male	94	27·2	99	28·0
Race or ethnicity				
Black	280	80·9	289	81·9
White non-Hispanic	39	11·3	42	11·9
Hispanic (regardless of race)	20	5·8	17	4·8
Other	7	2·0	5	1·4
Enrolment location**				
Federally qualified health centre	172	49·7	128	36·3
Senior assistance organisations	84	24·3	110	31·2
Academic primary care clinic	54	15·6	68	19·3
Dept of Health	15	4·3	24	6·8
Other	21	6·1	23	6·5
Primary insurance**				
Medicare	54	15·6	99	28·1
Medicare advantage	57	16·5	80	22·7
Medicaid	89	25·7	80	22·7
Private	51	14·7	41	11·6
Other, uninsured, VA, or unlisted	95	27·5	53	15·0
	Mean	sd	Mean	sd
Health status and healthcare utilisation				
Average HbA1c in pre-enrolment 6 months (%)**,†	5·65	0·34	8·32	2·16
Average BMI in pre-enrolment 6 months (kg/m^2^)**,‡	30·9	9·0	36·2	9·1
Average systolic blood pressure, pre-enrolment 6 months (mmHg)**,§	128·2	16·5	132·5	16·3
Average clinic visits, year post-enrolment**	11·5	12·0	17·3	15·6
Average emergency department visits, year post-enrolment	1·26	3·21	1·44	2·81
Average hospitalisations, in year post-enrolment**	0·29	0·74	0·69	1·47
	*n*	%	*n*	%
Programme utilisation				
Never spender	68	19·7	50	14·2
Sometimes spender	159	46·0	180	51·0
Frequent spender	119	34·4	123	34·8
	Mean	sd	Mean	sd
Baseline healthy food purchasing in month before enrolment				
Fruit and vegetable spending ($)	8·53	19·7	7·43	16·5
Fruit and vegetable expenditure share (%)‖	13·0	12·8	16·2	17·8
Fruit and vegetable diversity (unique items)‖	7·13	8·43	5·67	6·47

* Categories with statistically significant ANOVA or *χ* ^2^ test (*P* < 0·05), indicating difference between Never-Spenders, Sometimes Spenders and Frequent Spenders.

† *n* 61 and 287 for non-diabetes and diabetes subsamples, respectively.

‡ *n* 279 and 330 for non-diabetes and diabetes subsamples, respectively.

§ *n* 280 and 330 for non-diabetes and diabetes subsamples, respectively.

‖ *n* 154 and 175 for non-diabetes and diabetes subsamples, respectively.

** Categories with *P* < 0·01.

Continuous variables presented as Mean (sd), while categorical variables show column percentages (i.e. proportion within the subsamples with diabetes and without diabetes).

### Aim 2

#### Programme influence on healthy food purchasing

**Fig. 2:**
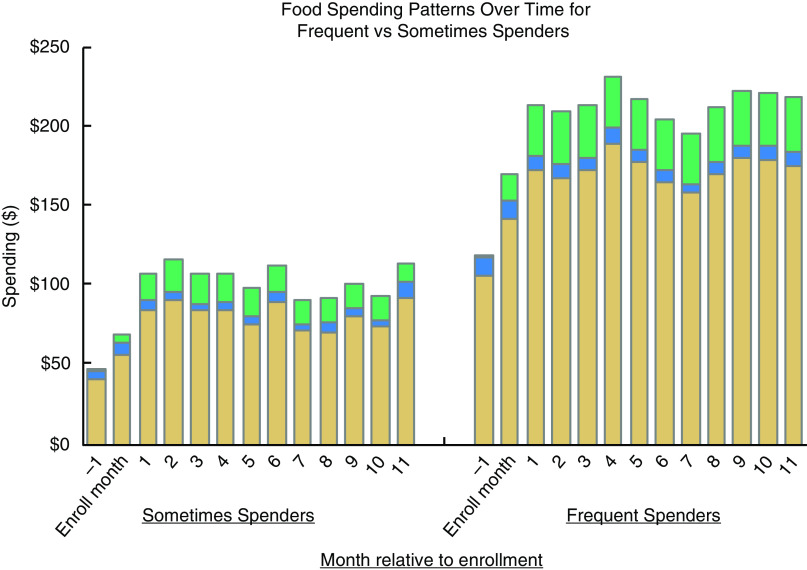
Food purchasing patterns for Produce Prescription Programme participants by utilisation level. Changes in food spending for participants month to month among those who were Frequent or Sometimes Spenders. See Supplemental Material for a table of data included in this figure. Two stacked bar charts showing spending patterns for Produce Prescription Programme Frequent Spenders and Sometimes Spenders. Each stacked bar has three segments to represent fruit and vegetable spending paid with Produce Prescription Dollars, fruit and vegetable spending paid with other methods and non-fruit and vegetable spending. Accompanying data are shown in table form in the Supplemental Material

Among all Frequent and Sometimes Spenders, fruit and vegetable purchasing increased over time, with Frequent Spenders experiencing the largest growth. Total food spending and fruit and vegetable spending increased the most within the first 2 months of enrolment. Produce Prescription Dollars accounted for the largest share of participants’ fruit and vegetable spending growth (Fig. 2). Higher programme utilisation was associated with higher fruit and vegetable purchasing (Table 3). In addition, the diabetes diagnosis covariate did not have a statistically significant effect on the full-sample regressions with our three healthy food purchasing outcome measures.

**Table 3:** Effects of produce prescription programme utilisation on fruit and vegetable purchasing, Durham, NC, 2018–2019

Key variable of interest	Effect on fruit and vegetable spending ($)			Effect on fruit and vegetable expenditure share (%)			Effect on fruit and vegetable diversity (unique items)†		
	Full sample	Diabetes sample	Non-diabetes sample	Full sample	Diabetes sample	Non-diabetes sample‡	Full sample	Diabetes sample	Non-diabetes sample
Frequent spender (relative to sometimes-spender)	$8·77**	$7·51**	$10·11**	3·3 %**	3·8 %*	3·2 %	2·52**	2·30**	2·99**
Monthly produce prescription dollars spent ($)	$0·80**	$0·80**	$0·79**	0·41 %**	0·40 %**	0·41 %**	0·23**	0·24**	0·23**

* Significant at *P* < 0·05 level.

** Significant at *P* < 0·01 level.

† Fruit and vegetable diversity is defined as the number of unique fruits and vegetables purchased in a month based on number of distinct item barcodes.

‡ Regression coefficient *P* = 0·065.

Full sample regressions include 581 participants; the diabetes subsample has 303 participants and the non-diabetes subsample has 278 participants.

#### Fruit and vegetable spending

Frequent Spender status was associated with higher monthly fruit and vegetable spending (*β* = $8·77, *P* < 0·001). The effect for participants with diabetes was $7·51 compared with $10·11 for the sample without a diabetes diagnosis. Each additional Produce Prescription Dollar spent was associated with higher fruit and vegetable spending (*β* = $0·80 per Prescription Dollar spent or $8·00 per 10 Prescription Dollars, *P* < 0·001). In both regression models for the full sample, non-fruit and vegetable spending, Hispanic ethnicity and other race were associated with higher fruit and vegetable spending.

#### Fruit and vegetable expenditure share

Frequent Spender status was associated with higher monthly fruit and vegetable expenditure share (*β* = 3·3 %, *P* = 0·007). The change in expenditure share was 3·9 % for participants with diabetes compared with 2·9 % for those without diabetes. The association was statistically significant for the diabetes subsample, but it did not reach statistical significance for the non-diabetes subsample. Monthly Produce Prescription Dollars spent was also associated with higher expenditure share (*β* = 0·41 % per Prescription Dollar or 4·1% per 10 Prescription Dollars, *P* < 0·001). In both regression models for the full sample, older age and Hispanic ethnicity were significantly associated with expenditure share. Non-fruit and vegetable spending and white race were associated with *lower* expenditure share.

#### Fruit and vegetable variety

Frequent Spender status was associated with increased fruit and vegetable variety, or the number of unique fruit and vegetable items in a month (*β* = 2·52, *P* < 0·001). The diversity increase was 2·30 items for participants with diabetes compared with 2·99 items for those without diabetes. Monthly Produce Prescription Dollars spent was also associated with increased variety (*β* = 0·23 or 2·3 items per 10 Prescription Dollars, *P* < 0·001). In the full sample, non-fruit and vegetable spending, older age, Hispanic ethnicity and other race were associated with increased variety.

### Aim 3

#### Health outcomes for participants with diabetes

For participants with diabetes, there were no statistically significant relationships between higher programme utilisation and diabetes-related outcomes, including BMI (*β* = 0·64, *P* = 0·54), HbA1c (*β* = -0·15, *P* = 0·53) and systolic blood pressure (*β* = -1·09, *P* = 0·51).

## Discussion

The current study evaluated a Produce Prescription Programme. Our primary interests were programme utilisation patterns and the programme’s effect on healthy food purchasing, but we also assessed diabetes-related health outcomes.

Higher programme utilisation likelihood after enrolment (Aim 1) was associated with being female and older. This is consistent with women often assuming primary responsibility for household shopping and meal preparation^(13,14)^. That seniors were more likely to utilise the Produce Prescription programme may be consistent with longer eligibility periods for elderly SNAP recipients^(15)^. Elderly SNAP recipients are more likely to remain enrolled in SNAP, though the overall proportion of eligible seniors who enrol in SNAP is lower than those of all other age groups^(16)^. This Produce Prescription Programme requires individuals to maintain SNAP eligibility, so younger individuals may have lost their ability to receive the Produce Prescription due to losing SNAP eligibility. It is also possible that two enrolment sites, which primarily serve seniors, engaged in more tailored outreach that led to higher utilisation.

Hospitalisations were negatively associated with any programme utilisation, and ED visits were negatively associated with more frequent programme utilisation. This is consistent with prior research showing a bidirectional relationship between food insecurity and hospitalisations, particularly in seniors. In one study, elderly individuals experiencing food insecurity were 40 % more likely to be having concurrent hospitalisation. That study found hospitalisation was associated with a 50 % higher chance of becoming food insecure in the future^(17)^. A hospitalisation can be a health and financial shock and a significant barrier to shopping for and cooking healthy food. Differences in programme utilisation by enrolment location suggest that there may have been site-specific variation in screening, communication strategies or instructional materials.

Higher programme utilisation (measured as being a Frequent Spender or higher Produce Prescription Dollars spent) was associated with higher fruit and vegetable spending, expenditure share and variety (Aim 2). This is consistent with other Produce Prescription Programmes, as well as non-clinic nutrition incentive programmes. For instance, a Produce Prescription Programme for patients with hypertension was associated with an increase of 3·3 to 4·9 daily fruit and vegetable servings and decreases in fast food consumption based on self-reporting^(18)^. In another study, 177 patients who received a clinic waiting room intervention to recruit people into the Southeast Michigan Double Up Food Bucks programme reported consuming an average of 0·65 more servings of fruits and vegetables at 3 months^(19)^. For monthly fruit and vegetable spending and variety, the positive effects associated with being a Frequent Spender were smaller for participants with diabetes compared with participants without diabetes. This may be related to the older average age of patients with diabetes in our sample. Older people face additional food access barriers, including living in food deserts and having functional limitations that may make it harder to physically go to a grocery store to utilise the programme^(20)^. This Produce Prescription Programme primarily covered uncooked foods, which could pose additional burden challenge for older participants with diabetes who may be affected by tremors, neuropathy, arthritis or lack of strength^(21)^.

There were no significant changes in diabetes-related health outcomes after up to 1 year of programme participation (Aim 3). Previous studies have been inconsistent in showing improved health outcomes despite healthier food consumption. A Detroit Produce Prescription Programme that gave 65 participants $40 per month for use at a clinic-located farmers market was associated with an average decrease in HbA1c from 9·54 to 8·53, but there were no changes in BMI or blood pressure^(22)^.

Our Produce Prescription Programme served a community that was disproportionately elderly, Black and low-income. These factors mean a $40 per month intervention may not sufficiently address multiple structural inequities such as racism and sexism. Nonetheless, this Produce Prescription Program was associated with increased healthy food purchasing including for participants with diabetes. Moreover, the positive health effects of good diet can take years to materialise. A systematic review found that several diets that emphasise fruits and vegetables are associated with better long-term cardiometabolic outcomes, including Mediterranean, Dietary Approaches to Stop Hypertension, vegetarian, Nordic and Portfolio diets^(23)^. Therefore, we should continue making healthy diets more accessible for people with the most socio-economic challenges.

### Strengths and limitations

The current study demonstrates the feasibility and strength of using two large existing data sets with food transaction and health data to measure Produce Prescription Programme impact on food purchasing and clinical health measures. Our study goes beyond prior Produce Prescription Programme research that has mostly relied on self-reported food security and survey-based food intake assessments.

Though we controlled for measured and unobserved participant differences through panel regression, participants were not randomised. For some participants, it was not possible to match all their health and food transaction data, possibly due to those participants being recruited from settings that are not part of Durham’s integrated EHR system (which covers the vast majority of those recruited at federally qualified health centre and academic clinic settings but not necessarily the participants enrolled through community organisations). This limits our ability to make strong claims about programme efficacy and causality. On the other hand, the nonrandomised design enabled assessment of utilisation predictors (Aim 1) to inform programme implementation.

The study was limited by imprecise food consumption measures. We only had access to transaction data from our partner grocery store chain, but not from other grocery stores, convenience stores, markets or other food assistance programmes. We lacked qualitative data on food preparation, consumption and/or waste. We also could not completely measure substitution and income effects due to programme participation. We expect a substitution effect towards more fruits and vegetables because they are effectively cheaper, but the income effect may increase total food consumption. In our study, we note total food spending increased, which means there is a theoretical possibility of not only more healthy food consumption but also more unhealthy food consumption. The dietary significance of competing increases in healthy and unhealthy purchases is difficult to quantify.

Due in part to programme features intended to minimise clinician burden, healthcare providers did not systematically ask patients about self-reported food security, as has been done in other studies. A study of Wholesome Wave’s multi-city Produce Prescription Programme was associated with improved food security score among 578 low-income households^(24)^. Also to minimise clinician burden, the Produce Prescription enrollers did not collect household size data, which prevents converting purchase data into food servings. However, we controlled indirectly for household size by including non-fruit and vegetable spending as a covariate in our food purchasing regressions. Nonetheless, our study’s strength lies in assessing real-world changes in participant food purchasing. This intervention likely increased food security, as indicated by the increases in nutritious food purchases and dietary diversity. However, without data on individual food intake or self-reported food security, we must be cautious about drawing strong conclusions about food quantity sufficiency.

This Produce Prescription Programme reached numerous people of colour and of lower socio-economic status, particularly by enrolling through a federally qualified health centre. While using SNAP for programme delivery enhances its efficiency and ease of use, this may have excluded Hispanic or Latino individuals, particularly those who are undocumented or have concerns about receiving government-administered assistance. This may partly explain the relatively low number of Hispanic participants in our sample. We found that despite there being fewer Hispanic or Latino Frequent Spenders, those who were Frequent Spenders actually had higher fruit and vegetable expenditures than other ethnic groups. However, we chose not to make strong conclusions about the effects of Hispanic ethnicity due to the relatively small number in our sample.

It is worth replicating and expanding this work: with larger samples, more healthcare settings (e.g. including paediatrics clinics), longer programme duration, analyses of different food groups including healthy and unhealthy foods and beverages and higher subsidy amounts to achieve clinically significant improvements in chronic disease management. Future Produce Prescription Programmes should include targeted outreach and potentially programme delivery without reliance on SNAP if it does not sacrifice efficiency. These efforts would enable serving a higher proportion of young and/or Hispanic people. For serving older individuals who may have additional co-morbidities and functional limitations, Produce Prescription Programmes should consider modalities involving home grocery delivery, online grocery shopping using SNAP and/or additional emphasis on healthy prepared foods – all of which have become more widespread as part of the retail food environment and food security interventions during the COVID-19 pandemic^(25,26)^. Following participants for a longer period may provide insight on long-term cardiometabolic outcomes. Future studies should also collect qualitative food consumption and food security data. It is also of interest to assess how Produce Prescription Programmes affect provider–patient relationships. For example, Produce Prescription Programmes have been associated with more frequent food and nutrition conversations with healthcare providers^(18)^. There is also a continued need for implementation research to improve healthcare provider and community organisation workflow.

### Implications for nutrition and public health

Produce Prescription Programmes can increase healthy food purchasing by bringing together clinics, community organisations and food retailers in novel ways. They are one of several forms of delivering ‘food as medicine.’ Food as medicine interventions connect health systems and food systems, including local food businesses^(27)^.

For healthcare providers, Produce Prescription Programmes provide a way to elevate the importance of healthy eating, raise awareness of local resources and further patient education^(28)^. For healthcare systems, Produce Prescription Programmes can improve diet-related illness management. Food as medicine interventions can decrease healthcare costs based on studies showing decreased health expenditures for those who receive SNAP and home meal delivery following hospitalisation^(29,30)^. Though it is difficult to determine causal direction, our results show a correlation between higher Produce Prescription Programme utilisation and fewer hospitalisations, and therefore, possibly healthcare cost reduction. Adding to evidence that Produce Prescription Programmes may reduce healthcare costs, a microsimulation study about Medicare and Medicaid beneficiaries estimated that a fruit and vegetable incentive could prevent 1·93 million CVD events and save $39·7 billion in healthcare costs^(31)^. Given the potential for health improvement and cost savings, health systems and insurance entities should consider making Produce Prescription Programmes medically reimbursable alongside other social drivers of health as part of the transition to value-based care^(32)^.

The current study reinforces the business case for grocery retailers to adopt Produce Prescription Programmes because of evidence that they expanded customer loyalty among programme participants, similar to other nutrition incentive programmes^(7)^. Additional analyses not elaborated upon in this paper showed that across all participants, total food spending at our partner grocery store chain grew after enrolment. This suggests that Produce Prescription Programmes may be associated with more regular shopping at the partner food retailer.

Our study shows how healthy food complements clinical care, medication, physical activity and other lifestyle changes. Produce Prescription Programmes make healthy food a major part of a patient’s health plan, yet their delivery through SNAP and grocery store loyalty programmes integrates participation seamlessly into the shopping experience. Produce Prescription Programmes can increase patient capacity to follow healthcare providers’ dietary advice with dignity and autonomy. Ultimately, food as medicine can enable health and food systems to deliver integrated services that improve healthy food access and chronic disease prevention and management.

## Supplementary material

For supplementary material accompanying this paper visit https://doi.org/10.1017/S1368980021001828. click here to view supplementary material
