# Supplementary material for: The impact of a produce prescription programme on healthy food purchasing and diabetes-related health outcomes
Source: Public Health Nutr. 2021 Apr 27;24(12):3945–55. doi: 10.1017/S1368980021001828 (PMC8369461; doi:10.1017/S1368980021001828)
Supplement: Supplementary file 1 [file S1368980021001828sup001.docx]

# Supplemental Material: Data for Figure 2, Food purchasing patterns for Produce Prescription Programme participants by utilisation level

| Sometimes Spenders | Month relative to enrolment month | Non-Fruit and Vegetable Spending | Fruit and Vegetable Spending using other pay method | Fruit and Vegetable Spending using Prescription Dollars |
| --- | --- | --- | --- | --- |
|  | **-1** | $41.91 | $4.19 | $0.18 |
|  | **Enrol month** | $57.12 | $7.26 | $5.06 |
|  | **1** | $85.05 | $6.67 | $16.18 |
|  | **2** | $91.37 | $4.83 | $20.32 |
|  | **3** | $84.50 | $4.71 | $19.00 |
|  | **4** | $85.12 | $5.12 | $17.13 |
|  | **5** | $76.21 | $5.11 | $17.06 |
|  | **6** | $89.34 | $6.79 | $16.39 |
|  | **7** | $71.49 | $4.48 | $15.63 |
|  | **8** | $71.35 | $5.25 | $15.33 |
|  | **9** | $81.04 | $4.66 | $15.61 |
|  | **10** | $74.10 | $4.20 | $14.83 |
|  | **11** | $92.95 | $9.47 | $11.35 |
| Frequent Spenders | **-1** | $106.28 | $11.39 | $0.21 |
|  | **Enrol month** | $142.50 | $11.84 | $15.89 |
|  | **1** | $172.42 | $9.62 | $31.72 |
|  | **2** | $167.60 | $8.70 | $33.08 |
|  | **3** | $173.04 | $7.30 | $33.02 |
|  | **4** | $189.86 | $9.23 | $32.18 |
|  | **5** | $178.18 | $7.11 | $32.22 |
|  | **6** | $165.64 | $7.84 | $31.76 |
|  | **7** | $158.41 | $5.75 | $32.20 |
|  | **8** | $170.08 | $8.02 | $33.77 |
|  | **9** | $180.85 | $7.81 | $34.30 |
|  | **10** | $178.80 | $9.98 | $32.66 |
|  | **11** | $175.71 | $9.24 | $33.40 |
